# Amygdalin: A Review on Its Characteristics, Antioxidant Potential, Gastrointestinal Microbiota Intervention, Anticancer Therapeutic and Mechanisms, Toxicity, and Encapsulation

**DOI:** 10.3390/biom12101514

**Published:** 2022-10-19

**Authors:** Hassan Barakat, Thamer Aljutaily, Mona S. Almujaydil, Reham M. Algheshairy, Raghad M. Alhomaid, Abdulkarim S. Almutairi, Saleh I. Alshimali, Ahmed A. H. Abdellatif

**Affiliations:** 1 Department of Food Science and Human Nutrition, College of Agriculture and Veterinary Medicine, Qassim University, Buraydah 51452, Saudi Arabia; 2 Food Technology Department, Faculty of Agriculture, Benha University, Moshtohor 13736, Egypt; 3 Department of Pharmaceutics, College of Pharmacy, Qassim University, Buraydah 51452, Saudi Arabia; 4 Department of Pharmaceutics and Pharmaceutical Technology, Faculty of Pharmacy, Al-Azhar University, Assiut 71524, Egypt

**Keywords:** amygdalin, antioxidant, anticancer, microbiota intervention, mechanisms, toxicity, encapsulation

## Abstract

Bioactive amygdalin, found in high concentrations in bitter almonds, has been recognized as a symbol of the cyanogenic glycoside chemical organic substance, which was initially developed as a pharmaceutical for treating cancer after being hydrolyzed to hydrogen cyanide (HCN). Regrettably, research has shown that HCN can also damage normal cells, rendering it non-toxic to the human body. Extreme controversy surrounds both in vivo and in vitro studies, making its use risky. This review provides an extensive update on characteristics, antioxidant potential, gastrointestinal microbiota intervention, anticancer therapeutic, mechanisms, toxicity, and encapsulation of amygdalin. Antioxidant, anti-tumor, anti-fibrotic, antiatherosclerosis, anti-inflammatory, immunomodulatory, and analgesic characteristics, and the ability to improve digestive and reproductive systems, neurodegeneration, and cardiac hypertrophy are just some of the benefits of amygdalin. Studies verified the HCN-produced amygdalin to be harmful orally, but only at very high doses. Although intravenous treatment was less effective than the oral method, the oral route has a dose range of 0.6 to 1 g daily. Amygdalin’s toxicity depends heavily on the variety of bacteria in the digestive tract. Unfortunately, there is currently no foolproof method for determining the microbial consortium and providing a safe oral dosage for every patient. Amygdalin encapsulation in alginate-chitosan nanoparticles (ACNPs) is a relatively new area of research. Amygdalin has an enhanced cytotoxic effect on malignant cells, and ACNPs can be employed as an active drug-delivery system to release this compound in a regulated, sustained manner without causing any harm to healthy cells or tissues. In conclusion, a large area of research for a substance that might be the next step in cancer therapy is opened up due to unverified and conflicting data.

## 1. Introduction

**Figure 1:**
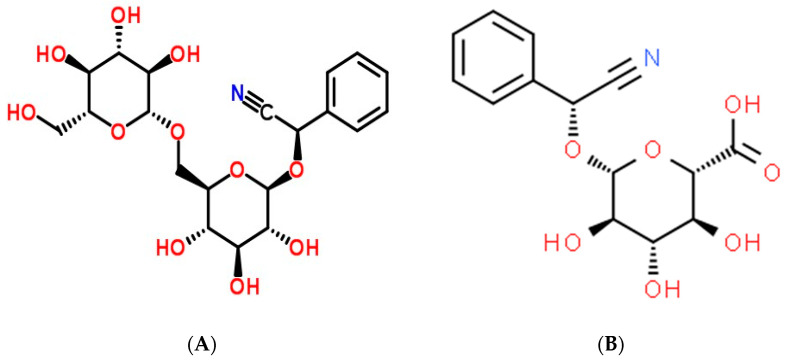
Amygdalin (**A**) and laetrile (**B**) chemical structures.

Amygdalin is a primary active pharmaceutical ingredient in almonds and is also commonly found in the seeds of Rosaceae species [1,2]. It is a naturally occurring substance that can be discovered in the seeds of many different plants [3]. The chemical formula for cyanogenic glycoside is C_20_H_27_O_11_ (Figure 1A), and it has a molecular mass of 457.42 g mol^−1^. Benzaldehyde, hydrocyanic acid, and two glucose molecules (D-mandelopnitrile-β-D-glucoside-6-β-glucoside), also known as Laeteil, make up amygdalin, a compound found in almond, apricot, and apple seeds [4]. Some of its qualities include anti-inflammatory, antibacterial, antioxidant, and immunomodulatory effects [3,5]. Hydrogen cyanide (HCN), a byproduct of amygdalin’s enzymatic hydrolysis, is dangerous, while amygdalin itself is harmless. Amygdalin’s positive benefits have been studied for decades, and the results have been consistently positive across a wide range of medical conditions, including but not limited to leprosy, colorectal cancer, asthma, bronchitis, and others [6,7]. Benzaldehyde, which is present in its molecule, makes it an analgesic [8]. However, the efficiency of its anticancer activity is still up for debate and requires additional research. This ability may be related to hydrocyanic acid emission during enzymatic hydrolysis [9]. Questions concerning amygdalin’s effectiveness as an anticancer treatment have been raised due to its toxicity to healthy cells and its limited pharmacokinetic properties. Inhibiting the growth of cancer cells by eliminating carcinogenic substances is assumed to be the primary anticancer activity. It is known as apoptosis [10], preventing the food supply to cancer cells, which reduces the prevalence of many types of cancer [11]. This review provides an extensive update on characteristics, antioxidant potential, gastrointestinal microbiota intervention, anticancer therapeutic, mechanisms, toxicity, and encapsulation of amygdalin. New data are expected to provide theoretical and practical clues for investigating amygdalin’s functional utility and applications regarding its anticancer functionality.

## 2. History and Structural Characteristics of Amygdalin as an Anticancer Therapy

Robiquet and Boutron-Charlard, two French chemists, discovered amygdalin (which was named “emulsin”) from bitter almonds in 1837 [12]. In 1845, it was tested as a potential cancer treatment in Russia, but it was quickly abandoned due to its high toxicity and poor efficacy. Germany rejected this cancer treatment in 1892. U.S. records show it was first used to cure cancer in the 1920s, but a subsequent clinical assessment by Dr. Ernst Krebs proved its danger to people. In the 1950s, a patent was issued for laetrile, an intravenous version of amygdalin that was said to be safe for human consumption. The National Cancer Institute (NCI) studied the quality of amygdalin products manufactured by Cyto Pharma of Mexico and found that both the oral and injectable forms did not exceed US pharmaceutical safety guidelines [7,13]. Therefore, it was rejected by the Food and Drug Administration (FDA). However, amygdalin had become one of the most popular anticancer approaches in the 1970s when it was reported that 70,000 American cancer patients were using it as a complementary and alternative therapy [14]. Twenty-three US states legalized its usage in 1980 for those with terminal cancer. Two FDA-approved clinical trials supported by the NCI in the 1980s showed that laetrile did not work.

The importation of amygdalin was outlawed in the United States and Mexico in 1987. However, laetrile is still produced and used as an anticancer treatment, particularly in Mexico [15]. Due to its cyanide toxicity, amygdalin is restricted to medical use in the United Kingdom [16,17]. Some studies equated amygdalin with vitamin B17, whereas others equated laetrile, a semi-synthetic injectable version of amygdalin, with vitamin B17 (Figure 1B) [18]. Commonly, people will use amygdalin, laetrile, or vitamin B17 interchangeably. This study will therefore stick to the research term employed in the primary sources. The amygdalin dose for in vivo research is provided in milligrams per kilogram of body weight (mg kg^−1^), while the in vitro dose is expressed in molar concentrations. Future amygdalin applications were considered in the context of amygdalin and other cyanogenetic glucosides as anticancer medications and potential anticancer agents [19].

## 3. In Vivo and In Vitro Antioxidant Potential of Amygdalin

Fruit stones have a high protein concentration in the kernel that can be used for other purposes. In addition to carbs and lipids, the embryo must store this substance for use throughout the earliest stages of development. Some fruit seed proteins are valued for their physicochemical features in addition to their nutritional benefits [20], benefits in complementary and alternative medicine [21], and also for serving as a rich source of bioactive peptides [22]. However, cyanogenic glycosides such as Amygdalin make it challenging to extract helpful protein from these renewable sources. Hydrolysis enzymes are generated when the plant cell structure is damaged, although amygdalin is non-toxic. By breaking down amygdalin, we obtain benzaldehyde, which has a bitter taste, and cyanide, which is toxic [23]. Numerous plants contain amygdalin, including almonds, pecans, cereals, and legumes. However, meals and beverages derived from these sources often have low levels of cyanide [24]. The legal limit of cyanogenic glycosides in foods is set by government legislation. For example, the European Food Safety Authority has set limits of cyanide levels of 35 mg kg^−1^ in alcoholic beverages, 5 mg kg^−1^ in canned stone fruits, and 50 mg kg^−1^ in nougat, marzipan, or their replacements or comparable goods [25].

**Figure 2:**
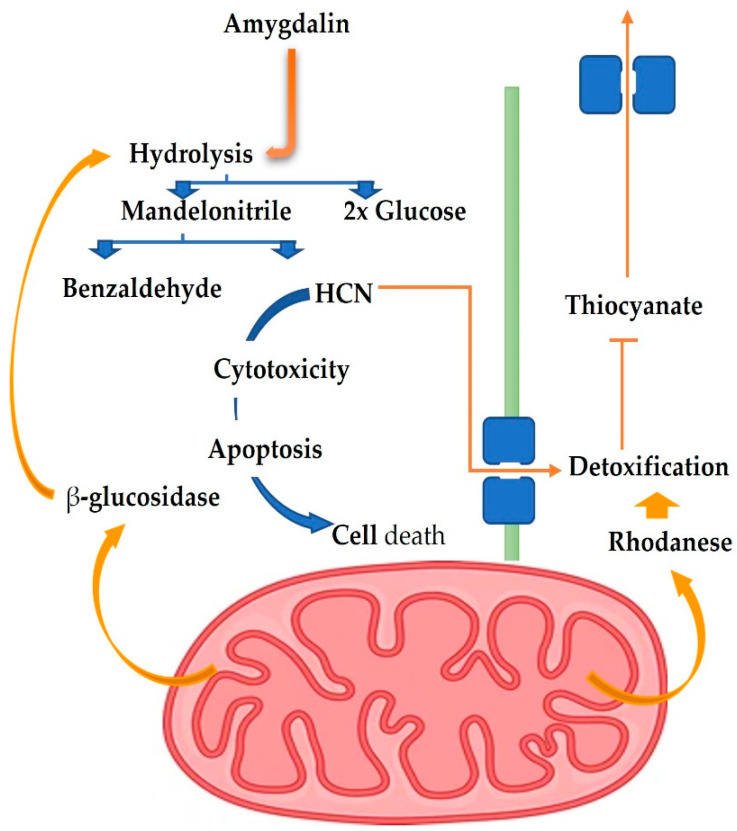
Controlling AMD in cancer and normal cells: the function of RHD and BGD [27].

Uniquely, apricot seeds include antioxidants, an Angiotensin I converting enzyme (ACE) inhibitor, and hypocholesterolemic peptides [26]. The antioxidant, antimicrobial, anti-inflammatory, and immune-regulating properties of amygdalin are in addition to its effectiveness against tumors [27]. The metabolizing enzymes rhodanese (RHD) and betaglucosidase (BGD) control their anticancer action in vivo. After treating Balb/c nude mice with amygdalin, Alwan and Afshari [27] found that the metabolic enzymes RHD and BGD played a crucial role in boosting the antigrowth of PC3 cancer cell lines. This provided insight into a potential mechanism of action for amygdalin, Figure 2. In contrast to high-dose amygdalin, which negatively affected the oxidative balance of male mice’s hepatic and testicular tissues, low- and medium-dose amygdalin had no such effect. Thus, amygdalin, in low doses, may restore oxidative equilibrium in mice [28]. Moreover, amygdalin exhibits significant antioxidant activity in the liver tissues and suppresses tunicamycin-induced endoplasmic reticulum stress in mice [29]. Amygdalin was isolated from *Prunus dulcis*, and its antioxidant and cytotoxic characteristics were investigated in vitro by Sushma et al. [30]. Multiple antioxidant experiments indicated that the *P. dulcis* amygdalin extract possessed strong antioxidant properties. The cytotoxic activity of amygdalin against HeLa cancer cells was found to be relatively high, and it possessed promising biological features overall. As its ethanolic extract of *C. horizontalis* branches contains natural vitamin E (0.76 mg 100 g^−1^ extract) and amygdalin, it was suggested by Sokkar et al. [31] that it may be a source of a natural antioxidant with hepatoprotective and hypolipidemic effects (0.11 mg 100 g^−1^ extract). Complete M.S. medium containing 2 mg L^−1^ BAP or 6 mg L^−1^ of naphthalene acetic acid stimulated vitamin E production *in vitro* cells, and a complete M.S. medium containing 4 mg L^−1^ kin or 2 mg L^−1^ 2,4 D stimulated amygdalin production.

## 4. The Microbiome and Oral Amygdalin Administration: Intervention with Gut Microbiota

The gastrointestinal tract (GIT) is home to roughly 10^14^ bacteria and 500 different species; the majority account for anaerobes. The intestinal microbiota receives nourishment from the hydrolysis of sulphates, amides, glucuronidases, esters, and lactones via enzymes such as sulfatase, esterase, α-rhamnosidase, β-glucosidase, and β-glucuronidase [32]. Certain lipids, proteins, carbohydrates, and peptidases are prebiotics that promote the host’s health by altering the makeup and activity of the gut microbiota [33]. The intestinal enzymes can be modulated with the help of probiotics such as Lactobacillus and Bifidobacterium, and harmful compounds can be absorbed or bound by these helpful bacteria. They can be consumed without risk to improve digestive health and protect against cyanide poisoning caused by amygdalin breakdown. It has been shown in vitro that fructooligosaccharides elevate Lactobacillus and bifidobacterial levels [34]. When ingested orally, however, medication is impacted by GIT enzymes in the lumen, gut wall, liver, and gut flora. Microbiome enzymes, gastrointestinal (GI) nucleases, lipases, transporters, peptidases, cytochrome P450, and proteases influence drug and nutrition metabolism. The drug concentration-time in the enterocytes is increased when blood flow to the intestinal mucosa is decreased. Brush border-attached glycosidases, which can cleave a glycosidic linkage, do not play a significant role in the metabolism of orally delivered medicines due to their requirement to bind to specific cleavage sites [35]. These two reasons explain why no germ-free animals are involved in cyanide poisoning after ingesting amygdalin. Two mammalian β-glucosidases, lactasephlorizin hydrolase and cytosolic-glucosidase, are located in the brush border mucosa of the intestine. These enzymes can convert bile and fatty acids into carcinogens [36,37,38]. Due to the low levels of enzyme activity in the small intestine, the undigested materials make their way to the colon, where they are broken down further by gut microbial enzymes. The β-glucosidases found in kernels are also responsible for hydrolyzing amygdalin [39]. Cyanide levels in the body are impacted by many factors, including the composition of gut bacteria [40]. The Bacteroides, in particular, have high glucosidase activity and release cyanide during the symbiotic digestion of amygdalin. Some β-glucosidases are found in the intestines, whereas others are found in bacteria. Antibiotics have been demonstrated to decrease gut flora, yet the administration of amygdalin resulted in the detection of just prunasin and no HCN. This reveals that amygdalin is metabolized by intestinal enzymes exclusively to Prunasin, which is then transported to the colon and entirely digested by microbial β-glucosidase [39]. It is challenging to assess the gut microbiota using current technologies, making it difficult to pinpoint an individual’s consortium and speculate on the toxicity and efficacy of individual doses [41]. Microbes from the genus Bacteriodetes were found to predominate in the gut microbiota of a polysaccharide ingested by termites, as reported by Zhang et al. [42]. It has been found that they contain a high concentration of the -glucosidase gene. Evidence suggests that Bacteriodetes, a bacterium found in the human colon, produces the β-glucosidase enzyme, a cellulose hydrolase essential for the breakdown of cellulose and hemicellulose. Studies of germ-free and control rats show that the gut microbiome stimulates the host to create more glucose and tri-acyl glycerol [43]. The presence of glucose in amygdalin may account for its high susceptibility to hydrolysis. The ratio of Bacteriodetes to Firmicutes decreases with increasing body weight in obese and non-obese persons [33,41,44]. Intestinal Bacteriodetes flourish in regions where the diet is high in fiber, such as Europe and rural Africa [41]. Diet, immunity, gut microbiota, and metabolism affect health and bacterial metabolism. Additionally, lactulose reduces the production of the Bacteriodetes enzyme β-glucosidase [33]. Supplementation with Lactobacillus and Bifidobacterium has been demonstrated in animal studies to reduce β-glucuronidase levels, indicating a decrease in -glucuronidase levels encoding Bacteriodetes [45,46]. Research shows that Lactobacillus and Bifidobacterium have significantly less β-glucosidase and β-glucuronidase activity than Bacteroides [47]. An induced intestinal cancers model showed reduced activity of Lactobacillus acidophilus and Lactobacillus GG enzymes. In contrast, the highest β-glucosidase activity was observed in *Bacteriodetes fragilis* [39,47]. Comparatively, Lactobacillus, Enterococci, Clostridia, and Enterobacteria tend to be more abundant in the guts of older persons [48]. Using comparative genomic research, Ravcheev et al. [49] demonstrated the presence of 269 glycoside hydrolases, such as glucuronidase and glucosidase genes, in *Bacteriodetes thetaiotaomicron.* According to research by Karlsson et al. [50], only closely related species of Bacteriodetes fragilis are found in the gut, and these bacteria are rich in carbohydrate-acting enzymes. However, research has revealed that lactic acid bacteria normally release this enzyme; therefore, increased bioavailability of dietary toxins and xenobiotics is expected [51]. These results need to be considered in order to identify settings where toxicity is common and identify potential approaches to adapt the system to deal with amygdalin poisoning. As a result of examining a person’s consortia, safer oral doses may be administered due to fecal tests.

The cyanide generated during hydrolysis of amygdalin is a CAM treatment because it binds to cytochrome oxidase c and a3, inhibiting respiration and DNA synthesis via reactive oxygen species, blocking cell nourishment, and finally inducing lysis [52,53]. Ingesting this does not have much of an impact on the digestive system. However, it can be helpful for parenteral delivery. The anaerobic gut bacteria generate large quantities of lactic acid via pyruvate fermentation [54]. This pH elevates glucosidase activity, which is harmful because amygdalin is hydrolyzed to cyanide and adds to the toxicity. Recent evidence from Blaheta et al. [39] suggested that cyanide is not responsible for the anti-tumor effects of amygdalin, as the compound retains these effects even in the absence of β-glucosidase. The inhibition of collagenase and hyaluronidase may protect against benign tumors by preventing the weakening of the intracellular matrix induced by these enzymes [55,56]. This combination of CAM therapies is commonly used. Vitamin C resources in the body are being depleted because of the high dosages. The synthesis of thiocyanate involves a rate-limiting stage in which cysteine plays a role [54]. The danger of cyanide poisoning is increased because of the physiological effects of vitamin C and the breakdown of amygdalin by gut bacteria [54]. Studies prove the same by Ward et al. [57]. The results of studies comparing the two treatments individually and together have been mixed. Some have blamed cyanide poisoning rather than cancer remission. They say this because the drug is taken orally rather than intravenously [58]. As β-glucosidase is not present and Rhodanese is present in intravenous techniques, HCN levels are reduced. Due to low Rhodanese levels in the GIT, detoxification from high oral doses is difficult [47]. Although cyanide poisoning might be challenging to diagnose, a quick and reliable prognosis can be obtained using arterial blood gas analysis [59]. Initial therapy after diagnosis involved using a cyanide antidote kit, which had to be discontinued due to toxicity concerns. Chromoturia and skin darkening in those with red complexions are the only clinically relevant adverse effects of cyanokit (hydroxocobalamin) [32,60]. Vitamin B12 has a higher binding affinity for cyanide, allowing it to chelate the toxin before it is excreted by the kidneys [61]. This characteristic makes it useful as a cyanide antidote [52]. An additional therapy option could be taking probiotics, which have been shown to reduce Bacteroides levels. However, the fact that Lactobacillus creates β-glucosidases was already common knowledge. HCN levels in cancer patients who consume a diet low in Bacteriodetes but rich in -glucosidase-producing Lactobacillus need more investigation.

## 5. Anticancer Therapeutic Effectiveness of Amygdalin

### 5.1. Anticancer Effects

Extensive studies have been conducted to confirm its therapeutic ability and protection for various cancer treatments [39,62,63,64,65,66,67,68,69]. They confirmed that amygdalin could exert its anticancer activity via apoptosis [63,70,71], preventing the growth of tumor cells [27,72,73,74] and tumor cell metastasis [75].

#### 5.1.1. Amygdalin as an Anti-Tumor Drug

Antibody-directed enzyme prodrug treatment is a systemic administration of antibodies against tumor antigens conjugated to enzymes (ADEPT). In the presence of the enzyme, the prodrug was locally delivered to the tumor, where it was transformed into a cytotoxic agent [76]. Amygdalin, a prodrug used to treat bladder cancer, can be broken by sweet almond β-glucosidase to create free cyanide. Malignant tumor cells might be eliminated locally, at the tumor location, without causing any systemic harm if this substance were activated there. It was thought to be a cytotoxic medication combined with the antibody to kill cancer cells. One of the potential targeted cancer therapies was using amygdalin and β-glucosidase in conjunction with the ADEPT system [77].

#### 5.1.2. Inhibiting Tumor Cell Growth

**Figure 3:**
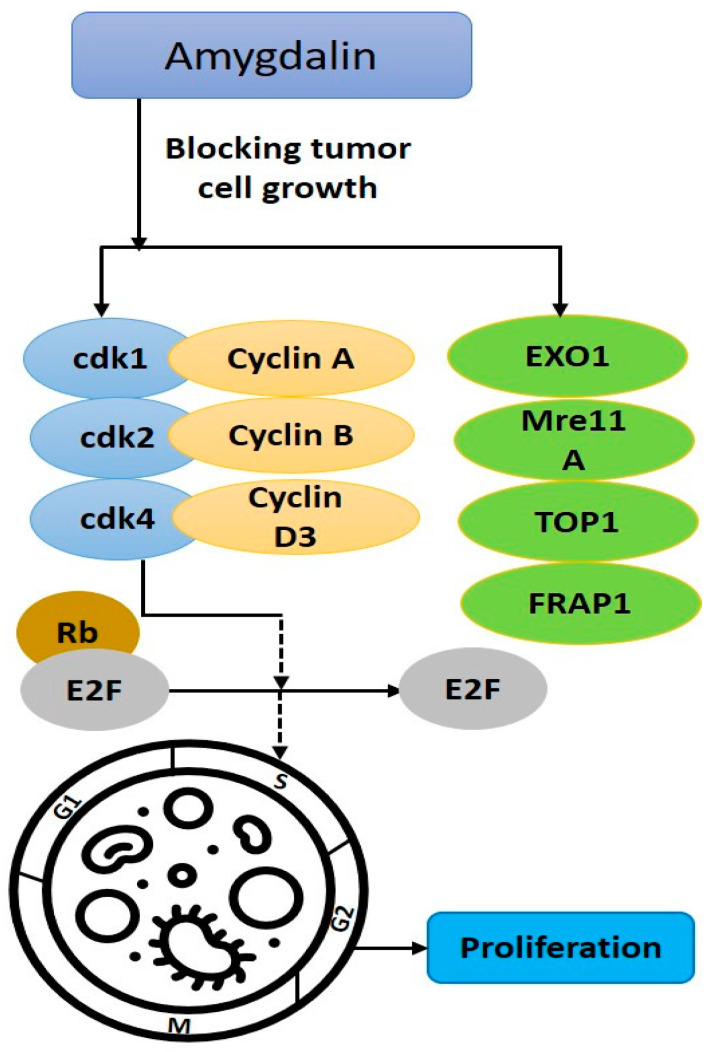
Amygdalin’s anti-tumor effects on cell proliferation.

Concentration-dependent tumor cell count reduction may occur with amygdalin (1–10 mg mL^−1^). The proliferation of LNCaP, PC3, and DU-145 cells was considerably stunted. Studies have indicated that amygdalin treatment suppresses the expression of proteins that govern cell division [78], Figure 3. Amygdalin (5 mg mL^−1^) reductions in exonuclease-1, ATP-binding loop subfamily F member 2, recombinant meiotic recombination 11 homolog A, topoisomerase I, and rapamycin-associated protein FK506 were seen in SNU-C4 human colon cancer cells. Cell cycle-related gene expression was inhibited, reducing SNU-C4 cancer cell growth [79]. The impact of amygdalin on the adhesion and motility of DU-145 and PC3 cancer cells was studied. Amygdalin inhibited the DU-145 cells’ chemotactic activity, migration, and adhesion more than the PC3 cells’. Amygdalin increased integrin two expressions in both cell lines. The amygdalin-induced downregulation of integrin 6 was specific to DU-145 cells, while the amygdalin-induced upregulation of integrin β1 was specific to PC3 cells. Since amygdalin inhibited six integrin expressions in DU-145 cells but not in PC3 cells, it appears that amygdalin treatment of some prostate cancer cells can block the metastatic dissemination facilitated by this integrin [80]. Amygdalin (1.25–10 mg mL^−1^) suppressed the growth and proliferation of UMUC3, RT112, and TCCSUP bladder cancer cell lines in a concentration-dependent manner, delaying cell cycle progression and G0/G1, reducing cyclin A and D2 and may stop tumor growth [81,82]. Oral squamous cell carcinoma (OSCC) cell line cytotoxicity and antiproliferative activity were observed with amygdalin (10–200 g mL^−1^), and KB cell viability was dose-dependently decreased [18]. Using the human breast cancer cell lines T47D and MCF-7, we found that amygdalin (at concentrations of 4, 8, 16, 32, and 65 mmol L^−1^) inhibited tumor growth in a dose- and time-dependent manner. Total glutathione production was six times higher in untreated MCF-7 cells compared with amygdalin-treated MCF-7 cells and two-and-a-half times higher in untreated T47D cells [83]. Amygdalin’s cytotoxic effect was measured in both cancerous breast cancer cell lines (MCF-7 and MDA-MB-231) and healthy human skin fibroblast cell lines [69]. Amygdalin reduced cell viability in both cell lines in a dose- and time-dependent manner, while it was non-toxic to the human skin fibroblast cell line at the same concentrations. Amygdalin (10 mg mL^−^^1^) has been shown to block the progression of numerous cancer cell lines, including U87-MG brain glioblastoma, MDA-MB-231 breast adenocarcinoma, MCF-7 breast adenocarcinoma, A-549 lung adenocarcinoma, and MRC-5 normal fetal lung fibroblasts [84]. Amygdalin (50 mg kg^−1^, i.v.) effectively reduced tumor weight and volume in colorectal xenograft model nude mice by 56.17% and 57.99% in vivo [85]. Amygdalin suppressed proteins and genes involved in controlling the cell cycle. Results from in vitro studies demonstrated that it controlled cdk and Akt-mTOR. More research is required to determine how it slowed cell growth to an insufficient point to trigger cell death [65].

#### 5.1.3. Reducing the Spread of Cancer Cells

**Figure 4:**
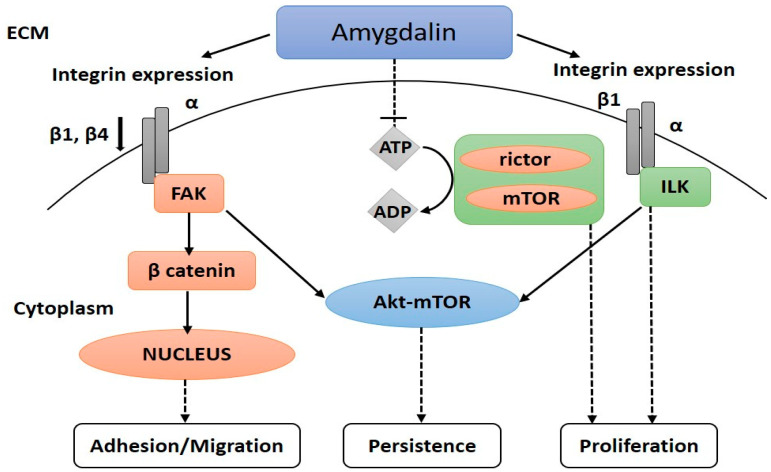
Amygdalin’s impact on Akt-mTOR signaling and β1 and β4 integrin expression. Lung cancer cell adhesion, migration, and proliferation are slowed when ILK and FAK expression is suppressed. The growth and proliferation of cancer cells are regulated by a phosphorylation cascade that includes the mTOR/Rictor complex, which amygdalin can block.

Pharmacological research has proven that the integrin-associated peptide focal adhesion kinase (FAK) is necessary for urothelial cancer cell motility [86], the activation of FAK was shown to be linked to cell adhesion, and the two processes were characterized [87]. Blocking FAK may halt cell migration in vitro [88] and minimize bladder cancer metastasis in rat models [89]. Amplification of β-catenin release by FAK is linked to tumor metastasis, which is related to integrin β1 and β4. The former can begin downstream signaling pathways, including Akt-mTOR, that mediate cell proliferation, adhesion, and metastasis by activating integrin-linked kinase (ILK). The latter enters the nucleus to control the expression of genes involved in tumor development and migration [90], Figure 4.

Amygdalin inhibited the proliferation of H1299/M and PA/M NSCLC cells by 15.6% and 25.1%, respectively; it reduced the expression of integrin 1, integrin 4, FAK, p-FAK, ILK, and –catenin in the cells while increasing the expression of E-cadherin [90]. At varying dosing levels, computational modeling and simulation investigated amygdalin’s effect on the PI3K-Akt-mTOR and Ras pathways. As previously mentioned, amygdalin was found to have a direct and significant effect on controlling PI3K-mTOR activity at threshold levels while having a secondary effect on the other cancer pathways. It follows that amygdalin acts as a down-regulator within a limited number of cancers and significantly helps fight against numerous human cancers [91]. Amygdalin (10 mg mL^−1^) inhibited cell adhesion in UMUC3, RT112, and TCCSUP bladder cancer lines, with the most significant effect seen in RT112 and TCCSUP. We see a general collagen binding and immobilization decline across all three cell lines. Amygdalin suppressed the expression of ILK and FAK activation and acted on integrin receptors in a cell-type-specific manner [81]. Amygdalin forms stable hydrogen bonds with the targeted targets, preventing ATP from entering the ATP-binding pocket of AKT1, FAK, and ILK, as revealed by computational simulations. The computational results provide crucial new information about the activity of amygdalin as a multi-target molecule in cancer metastasis and invasion. They expand our understanding of its action in many other ways [75].

Amygdalin reduced tumor volume in CD8F1 mice (BALB/c female hybrids = DBA/8 male producing spontaneous mammalian malignancies with an 80% incidence at ten months) in vivo at 1000 mg kg^−1^ and 2000 mg kg^−1^, i.p., reducing tumor volume by 54% and 33%, respectively. In addition, the rate of lung metastases might be lowered from about 90% to 22% with the help of amygdalin medication. [92]. Amygdalin has anti-tumor actions in cancer cell lines by inducing apoptosis and suppressing tumor cell proliferation and spread.

#### 5.1.4. Inducing Apoptosis

**Figure 5:**
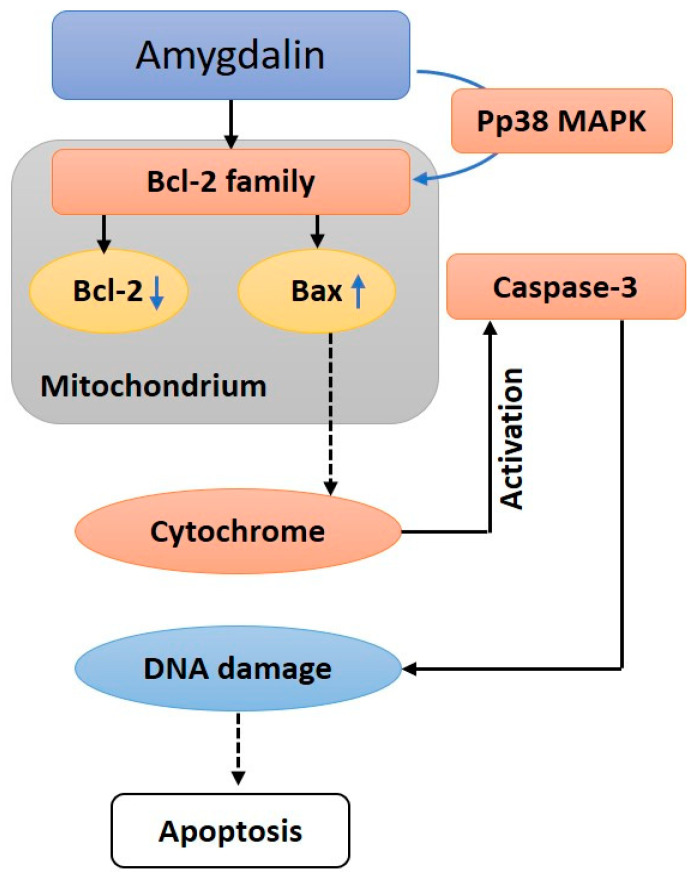
A schematic depicting how amygdalin induces apoptosis; amygdalin activates Pp38, which increases the level of BAX apoptotic proteins and decreases the quantity of Bcl-2 anti-apoptotic proteins and activates caspase-3.

Proteases of Bcl-2 and cysteine are involved in apoptosis. Apoptosis, or cell death, can be prevented by a protein called Bcl-2. The pro-apoptotic protein Bax is associated with high expression during apoptosis and subsequent cell growth [93,94]. Caspase-3 protease activation, initiated by the cytosolic replication of the Bax cytochrome C protein, is the primary mechanism of apoptosis [93,94]. Caspase-3 activation results in the cleavage of PARP, which initiates DNA strand breakage [8], Figure 5. The expression of Bcl-2 mRNA and protein might be suppressed by amygdalin (0.1–10 mg mL^−1^) in DU145 and LNCaP prostate cancer cells. In addition to increasing caspase-3 enzyme activity, it triggered apoptosis with typical morphological features [11]. When added to Hs578 T breast cancer cells, amygdalin (10–40 mg mL^−1^) drastically reduced Bcl-2 expression while increasing Bax expression and triggering the cleavage of caspase-3 and poly ADP-ribose polymerase [93].

Amygdalin (5–20 mg mL^−1^) may trigger apoptosis in a concentration-dependent manner by increasing pro-apoptotic Bax protein and decreasing anti-apoptotic Bcl-2 protein in SK-BR-3 cells (HER2 overexpressing human breast cancer cell line) [95]. By upregulating Bax expression and downregulating Bcl-2 and procaspase-3 levels, amygdalin (5–20 mg mL^−1^) has been demonstrated to trigger apoptosis in human cervical carcinoma (HeLa) cell lines. Furthermore, it dose-dependently decreased the survival rate of HeLa cells [11,96]. According to these results, amygdalin causes cells apoptosis by upregulating Bax expression and downregulating Bcl-2 [95]. Amygdalin as a possible therapeutic anticancer agent, supported by updated data, deserves development as a therapy for lung cancer [63], liver [70], as well as lowered cough-variant asthma-related airway epithelium apoptosis, inflammation, and epithelial-mesenchymal transition via inhibiting the TLR4/NF-kappaB signaling pathway [71].

### 5.2. Anti-Fibrosis Effect

Amygdalin’s antioxidant and anti-fibrotic actions on the liver and pancreatic fibrosis (Table 1) and pulmonary fibrosis have enhanced renal function in rats with chronic renal failure. Additionally, amygdalin may be useful in preventing fibrotic disorders such as renal interstitial fibrosis, liver fibrosis, and others [63,70,71,97].

#### 5.2.1. Modulating the Immune System

Human peripheral blood T-lymphocytes were stimulated to proliferate in vitro by phytohemagglutinin, and amygdalin (25–800 g mL^−1^) was found to be a potent stimulator of this effect. Increased secretion of IL-2 and interferon-y between 25 and 400 ng mL^−1^ has also been suggested [100]. At a dosage of 200 mg L^−1^, amygdalin (100–400 mg L^−1^) was shown to have the most significant influence on T-lymphocyte proliferation [105]. Immune cell proliferation was suppressed, immunosuppressive activity was exerted, and the time it took for renal transplanted rats to die was lengthened when amygdalin (10 mg kg^−1^, i.p.) was given to the animals in the living environment [100]. Inhibiting the local function of immune cells with amygdalin (5 mg kg^−1^, i.p.) significantly decreased the size of endometriotic foci. Many researchers now believe that immune cells play a crucial role in endometriosis’s onset and progression [106]. These results suggest that amygdalin can improve organ transplantation trials’ success rate by lowering immune cell proliferation during in vitro experiments. These seemingly contradictory results raise the intriguing possibility that amygdalin exerts a bidirectional regulatory function on the immune system.

#### 5.2.2. Efficacy in Preventing Atherosclerosis

In mice, amygdalin (1 mg kg^−1^, i.p.) dramatically decreased total cholesterol, triglyceride, low-density lipoprotein cholesterol, and matrix metalloproteinase-2 levels. Amygdalin inhibited plaque formation by inducing apoptosis in apolipoprotein E/mice [107] and stopping the body from making and using Toll-like receptors, slowing the progression of atherosclerosis. Serum levels of ALT, AST, and lipid transaminases were shown to be elevated when amygdalin (3 g kg^−1^, i.p.) was given to animals subjected to the Endoplasmic Reticulum stress model (ERSM) induced by tunicamycin injection [108]. Amygdalin (10 mg kg^−1^, i.p.) lowered lipid levels in mice deficient in the low-density lipoprotein receptor, including triglycerides, total cholesterol, and low-density lipoprotein [109].

#### 5.2.3. Contribution to the Reproductive Process

In vitro studies with porcine ovarian granulosa cells found that amygdalin (10 mg mL^−1^) induced the synthesis of steroid-regulating molecules (estradiol-17) [110]. Semen samples collected from Holstein bulls between the ages of two and three years old showed a significant decrease in hyaluronidase activity and spermatozoa motility when treated with amygdalin (0.4–2 mmol L^−1^) [111]. In vitro, amygdalin increased testosterone levels in both testicular homogeneous tissue and blood by increasing Na+ −K+ −ATPase and superoxide dismutase concentration. In mice exposed to lead acetate, it reduced the prevalence of deformed sperm and restored spermatogenic activity [112]. Amygdalin was also assumed to be a significant pharmacological component of the Japanese ovary-modulating Keishibukuryo-gan [113].

#### 5.2.4. Neurodegeneration Improvement

Amygdalin (2.5–20.0 mmol L^−1^) increased neurite outgrowth in nerve growth factor-producing PC12 cells from rat pheochromocytomas, and it shielded these cells from the neurotoxicity induced by 6-OHDA by upregulating calreticulin synthesis [114]. It was found to be an effective neurotrophic agent (0.003–0.020 mmol L^−1^) and could promote extracellular signal-regulated kinase 1/2 activation in PC12 cells [115]. Results suggest amygdalin’s role in protecting cells from neurotoxicity and highlight its potential application in treating neurodegenerative disorders.

#### 5.2.5. Anti-Renal Interstitial Fibrosis

Renal interstitial fibrosis is caused by an excessive extracellular matrix (ECM) accumulation and by the proliferation of kidney fibroblasts (KFBs). Renal fibroblasts can be transformed into myofibroblasts by the action of the main cytokine-transforming growth factor-1 (TGF-1) [116]. The proliferation of human KFB cells has been demonstrated to be inhibited by amygdalin in a concentration-dependent way. Amygdalin (3 and 5 mg kg^−1^, i.p.) decreased tubulointerstitial lesions in rats with unilateral ureteral obstruction in vitro. [102]. Results show that amygdalin effectively blocks the advancement of renal fibrosis by suppressing the activation of interstitial fibroblasts in cultured kidneys.

#### 5.2.6. Anti-Liver Fibrosis

Key producers of liver matrix components (ECM) and regulators of ECM production and secretion, hepatic stellate cells (HSCs), were once thought to have a major role in liver structure and function. Abnormal synthesis and accumulation of extracellular matrix (ECM) proteins define the pathological liver fibrosis process [114]. Important in mediating HSC-induced hepatic fibrosis is transforming growth factor beta (TGF-β) [103]. As a downstream effector of TGF-β, connective tissue growth factor (CTGF) plays a pivotal role in mediating tissue remodeling and fibrosis [70]. Both are critical fibrotic factors that contribute to the development of liver fibrosis. CTGF and TGF-β levels in HSCs were lowered in mRNA and protein expression when treated with amygdalin (200 g mL^−1^) [103]. In a separate study, amygdalin was found to strongly suppress the expression of platelet-derived growth factor (PDGF), insulin-like growth factor mRNA, and PDGF (10^−5^ mol L^−1^). According to this research, amygdalin has shown promise as a potential new treatment for liver fibrosis. An in vitro analysis of the biochemical and molecular mechanisms responsible for apricot extract’s (AE) and amygdalin-containing fraction’s (ACF) therapeutic actions on DMBA-induced liver carcinogenesis was conducted. These results showed that AE and ACF effectively prevent DMBA-induced hepatocarcinogenesis and that the major proteins implicated in proliferation, angiogenesis, autophagy, and apoptosis are viable molecular targets with hepatothrapeutic potential [70]. Amygdalin’s effect on hepatocellular carcinoma (HepG2) cell lines has been studied both with and without zinc treatment. Treatment of HepG2 cell lines with amygdalin plus 20 mol zinc or amygdalin plus 800 mol zinc resulted in a much greater apoptotic impact than with amygdalin alone. For HepG2, adding zinc greatly improved amygdalin’s efficacy [117].

#### 5.2.7. Anti-Pulmonary Fibrosis

In a rat model of bleomycin-induced lung fibrosis, amygdalin (15 mg kg^−1^, i.p.) reduced collagen expression of types I and III. Type III collagen was more susceptible to inhibition by amygdalin than type I collagen. This may have something to do with the extracellular matrix’s maturation timing during the fibrotic process. In silicosis, it may also inhibit collagen production in the lungs [101]. Further research found that in rats with SiO_2_-induced pulmonary fibrosis, amygdalin (48 mg per rat, i.p.) significantly decreased serum ceruloplasmin and lung collagen [118].

#### 5.2.8. Anti-Inflammatory Effect

Macrophages are critical components of the immune system’s innate defenses because they stimulate the production of inflammatory cytokines such as tumor necrosis factor-alpha (TNF-α), interleukin (IL)-1, IL-6, and IL-12. They are crucial in reducing inflammation, which is a critical factor in many disorders [94,119,120]. Amygdalin might prevent mouse BV2 microglial cells from producing prostaglandin E2 and cyclooxygenase COX-1. Inflammatory illnesses are significantly impacted by overactive p38 MAPK/NF-kB signaling [121]. Mouse peritoneal macrophage IL-17A, IL-23, chemokine 2, and chemokine 5 mRNA expression and p-p38 protein levels were all significantly decreased after exposure to amygdalin (50 mmol L^−1^) (RAW264.7) [122]. Amygdalin-induced acute lung damage lowered lipopolysaccharide levels in vivo (0.5–2 mg kg-1, i.p). (LPS). TNF-, IL-1, IL-6, and NF-kB levels dropped. Amygdalin may reduce TNF-, SIAM-1, and TNF- in rats with type II collagen-induced arthritis [103]. The results show that amygdalin inhibits inflammation by lowering levels of inflammatory markers and regulating the p38 MAPK/NF-kB signaling pathway.

#### 5.2.9. Pain-Relieving Effect

Amygdalin decreased IL^−1^ and TNF- mRNAs and regulated LPS-activated RAW 264.7 cells. Amygdalin (0.005 mg kg^−1^, i.m.) may help carrageenan-induced arthritic rats [123]. The peak of amygdalin’s analgesic efficacy after oral administration (100 and 300 mg kg^−1^) occurred between 1.5 and 2 h. The drug’s effects diminished over the next 4 h without showing signs of tolerance [124]. These results indicate the potential use of amygdalin as an analgesic due to its anti-nociceptive and anti-inflammatory properties. However, its analgesic and anti-inflammatory properties were examined [118]. The most important references discussing its painkilling and anti-inflammatory properties are listed in Table 2.

## 6. Anticancer Mechanisms of Amygdalin: A Molecular Approach

Amygdalin was not always seen as a risky cancer drug. It was well-received by proponents of alternative medicine due to the widespread belief that its hydrolysis would kill only cancer cells by generating toxic HCN. Unfortunately, reviewed studies by Liczbiński et al. [128] show that HCN is also produced in normal cells, so it may not be safe for humans. Even so, researchers have studied this compound’s potential cancer-fighting effects. Through upregulation of pro-apoptotic Bax and caspase-3 expression and downregulation of anti-apoptotic BcL-2protein expression, amygdalin has been shown to induce apoptosis in vitro. Amygdalin has been demonstrated to block the Akt-mTOR pathway (Figure 4), which may lead to a decrease in cancer cell metastasis by preventing cancer cells from adhering to one another. This effect has been shown in breast, lung, and bladder cancer. Amygdalin was also found to boost p19 protein expression in kidney cancer cells, preventing the progression of cells from G1 to S-phase and reducing cell proliferation. Recent research suggests that amygdalin has an anti-inflammatory effect by reducing the expression of pro-inflammatory cytokines such as pro-IL-1β by inhibiting the NF-kappaB and NLRP3 signaling pathways. The amygdalin-induced changes in TGF-β/CTGF pathway expression of follistatin and anti-fibrous activity that stimulated muscle cell development were also documented. Potentially useful in the fight against many forms of cancer, this chemical is being studied for its potential role in therapy. TGF-beta/Smad signaling is involved in bone fractures. However, amygdalin has potential as an alternative treatment [129].

## 7. Toxicological Effects of Amygdalin

After oral administration, amygdalin is broken down by amygdalin lyase into prunasin and glucose, both of which are non-toxic. Prunasin lyase catalyzes the hydrolysis of prunasin, resulting in glucose and mandelonitrile. Then, OH-nitrile lyase breaks down the mandelonitrile into hydrogen cyanide and benzaldehyde [130]. HCN impedes cell respiration, and it also interacts with cytochrome oxidase. The cyanide ion (CN-) is formed during the metabolism and absorption of hydrocyanic acid, inhibiting the body’s capacity to use oxygen by interfering with the respiratory electron transfer mechanism. Amygdalin is harmful because it causes cells to undergo hypoxia and lactic acidosis [131]. The daily oral dose of HCN (1 g of amygdalin released 59 mg of HCN) for adults (50–60 kg) demarcated by WHO is 0.6–0.72 g [118]. It is not yet known whether or not 500 milligrams of totally hydrolyzed amygdalin releases 180 milligrams of HCN, which is lethal to adults. Serum levels of 20 g HCN/dl were found hazardous, while levels of 300 g HCN/dl in the serum and 500 g HCN/dl in the blood were fatal [118]. In cases of amygdalin overdose, this might be caused by a toxic metabolite, and it would provide rough loci for HCN poisoning [39].

According to previous findings, consuming significant quantities of bitter almonds has been linked to cyanide poisoning (0.5–3.5 mg kg^−1^) in children and adults, according to previous findings [132]. In addition, the chance of developing severe adverse effects triggered by cyanide poisoning was elevated when amygdalin was taken orally. Consuming vitamin C simultaneously as amygdalin increases the risk, speeds up the process by which amygdalin is converted to cyanide, and heightens the toxicity [53]. Vomiting, shortness of breath, nausea, irritability, headache, tachycardia, disorientation, loss of consciousness, and seizures are all outward signs of cyanide poisoning. Unresponsiveness, hypotension, arrhythmia, cardiac arrest, respiratory failure, convulsions, cyanosis, an unusual or bitter almond odor, and cherry red skin are all symptoms of cyanide poisoning. There were 26 deaths attributed to serious cyanide poisoning in research that analyzed 65 studies (there were 52 case reports and 13 series). Respiratory failure (96%) was the most commonly cited clinical symptom of death, followed by unresponsiveness (92%), hypotension (85%), cardiac arrest (58%), and bradycardia (5%). Demyelination of peripheral nerves, optic neuropathy, deafness, and Parkinson’s disease are only a few neurological issues that may result from amygdalin poisoning [133].

The mean lethal dose (LD_50_) and the approximate lethal dose was 9279.50 mg kg^−1,^ and 1000~2000 mg kg^−1^ in prebrewed *Armeniacae semen* extracts in female and male rats, respectively [79]. For mice, rabbits, and dogs, the highest tolerable intravenous or intramuscular dose of amygdalin was 3 g kg^−1^, while the lowest tolerable intragastric dose was 0.075 g kg^−1^ [118,134]. Results from the severe toxicity testing showed that the oral direction method was 40 times more harmful than the venous injection [118]. Animal trials using 500 mg g kg^−1^ of amygdalin administered intravenously revealed no fatalities, while the same amount administered intragastrically resulted in an 80% mortality rate within 48 h. Symptoms of systemic toxicity, such as changes in atrial premature beats, ECG T waves, and gastrointestinal distress, may result after taking 4 g of amygdalin orally once daily for 15 days or intravenously for 30 days [53,135]. Toxicity may be avoided at doses between 0.6 and 1.0 g kg^−1^, or it may disappear after the substance is no longer present [7].

It has also become well known that different routes of amygdalin administration use different things on the receiver, adding to the growing body of reports on the topic. After oral administration, amygdalin requires prehydrolysis via brush border β-glucosidase, leading to a low blood concentration of amygdalin, an increase in cyanide content, the conversion of amygdalin into Prunasin, and the detection of just the precursor of its metabolite prunasin in plasma [39]. The amygdalin concentration in the blood was very high after the intravenous injection, and very little cyanide was available [136], and a precursor to amygdalin could be seen in the plasma. Although amygdalin was primarily eliminated in its unique form via urine following venous infusion, the peak defecation rate occurred 2 h after infusion, and the cumulative flow degree by 24 h was 79.6% [137]. Research suggests that amygdalin administered intravenously avoids breakdown by digestive enzymes and produces no cyanide toxicity in combination with its ineffectiveness.

Similarly, there may be notable changes in blood cyanide levels after oral treatment. Antioxidant gene expression and the reduction of oxidative damage in mice were studied in response to varying dosages of amygdalin. Amygdalin at doses of 50 and 100 mg kg^−1^ did not cause toxicity in male rats’ hepatic and testicular tissues. However, a dose of 200 mg kg^−1^ did harm the oxidative balance in mice [28]. In vitro studies using the comet test examined amygdalin’s genotoxic and antigenotoxic effects on human peripheral blood cells. Antigenotoxic activity against oxidatively damaged DNA and antioxidant effects on human lymphocytes demonstrated by Erikel et al. [138] suggest that amygdalin is not genotoxic.

## 8. Microencapsulation and Bioavailability

The anti-cancer effects of amygdalin are most clearly seen in its ability to induce apoptosis in cancer cells, inhibit the growth of cancer cells, and degrade carcinogenic components [10], reducing the prevalence of cancer by preventing nutrients from reaching cancer cells [11]. Many types of cancer, leprosy, emphysema, vitiligo, asthma, and bronchitis have all benefited from its administration [6]. Though promising as an anticancer agent, amygdalin has been called into question due to its toxicity to healthy cells and troublesome pharmacokinetic features. While amygdalin has been utilized occasionally, no studies have been conducted using drug delivery nanocarriers. Amygdalin’s medicinal effectiveness and adverse effects can be improved through encapsulation. Researchers are developing and studying nanocarriers for various applications, one of which is a “smart medicine delivery system” [139]. Nanoparticle drug delivery has considerably improved many aspects of drug administration, including therapeutic effect duration, drug stability, parenteral or enteral administration, cumulative drug penetration, suppression or elimination of drug metabolism, cellular efflux, and excretion [139]. Nanoparticles (1–200 nm) have the drugs either encapsulated inside or adsorbed on their surfaces. Nanoparticles can be manufactured not just from metals but also from natural biopolymers, polysaccharides, synthetic polymers, and lipids. Since colloidal polymeric nanoparticles are so small, they may be able to be aligned in a way that allows for controlled and sustained drug release [140]. Their biodegradability, nontoxicity, biocompatibility, low cost, and abundant availability make them attractive candidates for drug-delivery systems [141].

**Figure 6:**
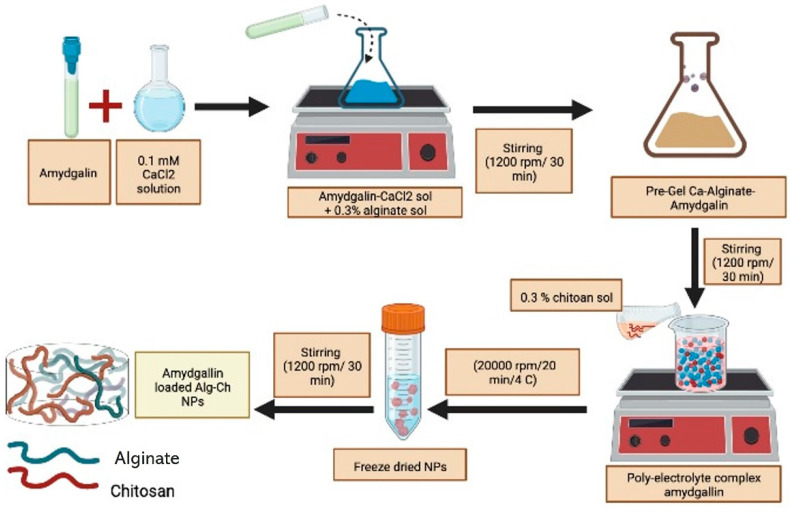
An illustration showing how anionic and cationic ACNPs loaded with amygdalin are made.

Amygdalin has been viewed skeptically despite its cancer-fighting potential due to its cyanide group. Amygdalin was encapsulated and tested for delivery to cancer cells by studying the potential of alginate-chitosan nanoparticles (ACNPs) as drug-delivery systems, Figure 6.

**Figure 7:**
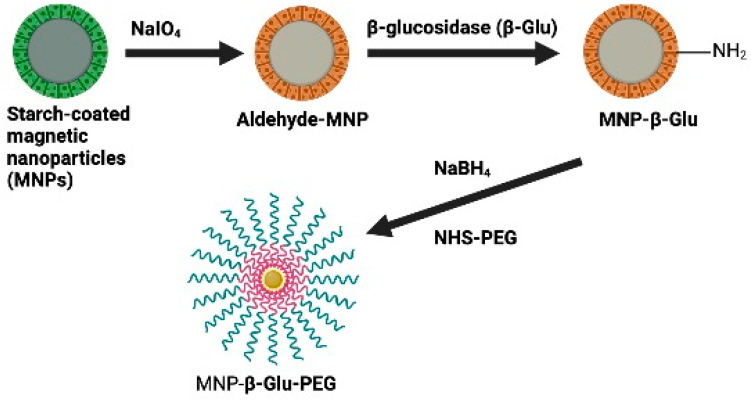
Schematic diagram of preparation process of MNP-β-Glu-PEG.

To further investigate charge dependence on drug delivery and cytotoxicity, amygdalin-acNPs loaded were created with anionic and/or cationic outer layer. The nanoparticles demonstrated ten hours of continuous drug release and high bump rates in neutral and slightly acidic conditions [139]. ACNPs showed a pH-dependent increase in swelling and a sustained release drug form (3.1, 5.0, 7.4). They could quickly get through the shear flow stress of 10 dynes cm ^−2^. This motivates additional in-vivo research on cancer tumor models with the produced ACNPs. As can be seen in Figure 7, Zhou et al. [142] showed that starch-coated magnetic nanoparticles (MNPs) could be progressively connected with β-glucosidase (β-Glu) and polyethylene glycol (PEG). Potentially useful in the fight against prostate cancer is physically directed enzyme/prodrug therapy (PEG). PEG modification has decreased nanoparticle formation in the liver and spleen to increase the accumulation of β-Glu-loaded nanoparticles on specific tumor tissue subjected to an external magnetic field.

## 9. Future Perspectives and Conclusions

Traditional remedies for leukoderma, leprosy, bronchitis, nausea, and cough included amygdalin. In vivo and in vitro have confirmed its pharmacological effects, which include antioxidant, anti-tumor, anti-fibrotic, anti-inflammatory, analgesic, immunomodulatory, and anti-atherosclerotic effects, peptic system and reproductive effects, neurodegeneration, and myocardial hypertrophy, lowering blood glucose. Despite this, our present understanding of the molecular mechanisms behind amygdalin’s activities is limited, as most existing research has focused on the compound’s pharmacological efficacy and toxicity. The results of the studies are controversial, making it risky to use as a treatment. Estimates of amygdalin’s target-organ toxicity and systemic information on the drug’s pharmacokinetics are lacking. Therefore, further state-of-the-art research on its possible therapeutic effects, side effects, and toxicity is required in the future. Recent studies have focused on the effects of this factor on cardiac hypertrophy, blood glucose, inflammation, digestion, neurodegeneration, and reproduction, but further investigation is needed to acquire a complete picture of its role. These investigations, while cumulative, are far from complete. Recent studies have shown that oral ingestion of amygdalin is more hazardous than intravenous administration. Its mechanism of action, toxic dose, and dependence on the gut consortium are all mysteries. Yet, in vivo studies of amygdalin with drug delivery nanocarriers have been limited. Recent studies using amygdalin-loaded ACNPs and an MDEPT approach based on amygdalin/-Glu show promise for potential use in future clinical trials for cancer treatment. For this reason, research into its encapsulation and anti-cancer efficacy in this form should be prioritized to improve therapeutic benefits and reduce the adverse effects of amygdalin.

## Figures and Tables

**Table 1:** Evidence for amygdalin’s anti-fibrosis effects: organized reviews and meta-analysis of the primary literature.

Model	Dose/Method/Period	Activity	Refs.
KFB	100 μg mL^−1^; culture; 48–72 h 80 μg mL^−1^; culture; 48–72 h 80 μg mL^−1^; culture; 24–48 h	Collagenase Activity Types ↓ Type-collagen ↓ Apoptosis of KFB ↑	[98]
Tracheal injection of 50 mg/mL SiO^2^ suspension 1ml, SD rats	48mg mL^−1^; i.p.; 1 month	Creating collagen in the lungs ↓ Blood ceruloplasmin ↓	[99]
KFB	100 mg L^−1^; culture; 48 h	Significantly reduces KFB cell proliferation.	[100]
Tracheal injection of 5 mg kg^−1^ bleomycin, Wistar rats	15 mg kg^−1^ body weight; ip; 28 days	collagen types I and III ↓	[101]
KFB	25, 50, 100, 200, 400 and 800 μg mL^−1^; culture; 48 h	TGF-β1 ↓ KFB proliferation is dose-dependently inhibited.	[102]
Wistar rats undergo unilateral ureteral obstruction	3 mg/kg, 5 mg kg^−1^; i.p.; 7,14 and 21 days	Renal interstitial lesion severity markedly decreased by day 21.	[102]
HTC-T6 cells	200 μg mL^−1^; culture; 48 h and 72 h	TGF-β ↓ CTGF ↓	[103]
HTC-T6 cells	10^−5^, 10^−4^ and 10^−3^ mol L^−1^; culture; 48 h	PDGF ↓ IGF ↓	[103]
CP model rats (injecting DBTC into the right caudal vein)	An intravenous injection of 10 milligrams per kilogram of body weight once daily for the first three days, then every other day for 28 days.	Pancreatic fibrosis, Acinar destruction, α-SMA, PDGF-BB, TGF β-1, and ET-1 ↓ CGRP ↓	[104]

**Table 2:** Provides a summary of the primary references dealing with its analgesic and anti-inflammatory effects.

Model	Dose/Method/Period	Activity	Refs.
Glial cell type BV2	1, 10, 100, 1000 μg mL^−1^; culture; 24 h	COX-2 mRNA, iNOS mRNA the synthesis of prostaglandin E2 the production of nitric oxide	[121]
Cell line RAW 264.7	1, 10, 100 mmol L^−1^; culture; 6 h	At a concentration of 1 mM, TNF-α and IL-1β mRNA Amygdalin does not inhibit TNF-α and IL-1β mRNA expression in a dose-dependent manner.	[123]
SD male rats with Carrageenan-induced arthritis pain model	0.005, 0.05 and 0.1 mg kg^−1^; im; 8 h	At a concentration of 0.005 mg/kg, Fos, TNF-α and IL-1β; However, no analgesic effect of amygdalin was observed at doses greater than 0.005 mg/kg	[123]
Modeling pain via plantar injection of formalin in SD male rats	0.1, 0.5, 1.0 and 10.0 mg kg^−1^; Plantar injection	C-Fos, TNF-α, IL-1β Laetrile reduces pain in a dose-dependent manner in a dose range of less than 1 mg/kg.	[123]
Type II collagen-induced CIA model, Wistar rats	120 mg kg^−1^; gavage; 28 days	TNF-α and sICAM-1	[125]
The BALB/c Mice	0.5, 1 and 2 mg kg^−1^; ip; 7 h	NF-κB Reduced pulmonary edema in a dose-dependent manner.	[126]
RAW264.7 cells	6.25, 12.5, 25, 50, 100, 200, 400 µmol L^−1^; culture; 24 h	IL-17A, IL-23, CCL2 and CCL5 mRNA p-p38 the viability of RAW264.7 cell	[127]

## Data Availability

Not applicable.

## Abbreviations

ACNPs, alginate–chitosan nanoparticles; ADEPT, antibody-directed enzyme prodrug therapy; Bax, Bcl-2 Associated X protein; Bcl-2, B-cell lymphoma-2; CAM, Conventional and alternative medicine; CDK, cyclin-dependent kinases; CTGF, connective tissue growth factor; ECM, extrac ellular matrix; ERSM, Endoplasmic Reticulum stress model; FAK, focal adhesion kinase; FDA, Food and Drug Administration; GIT, gastrointestinal tract; HCN, hydrogen cyanide; HSC, hepatic stellate cell; i.m., intramuscular injection; i.p., intraperitoneal injection; i.v., intravenous injection; IL, interleukin; ILK, integrin-linked kinase; KFB, kidney fibroblast; LD_50_, the median lethal dose; NCI, National Cancer Institute; NF-κB, nuclear factor kappa beta; PDGF, platelet-derived growth factor, TGF-β1, transforming growth factor-β1; TNF-α, tumor necrosis factor-α; US, United States.
